# Combined medical-interventional approaches for the management of complex fungal balls: a case series as a viable alternative in non-surgical patients

**DOI:** 10.1177/17534666241255203

**Published:** 2024-05-24

**Authors:** Elaine Dumoulin, Christina S. Thornton, John H. MacGregor, Alain Tremblay, Chrystal Chan, Paul R. MacEachern, Margaret M. Kelly, Ranjani Somayaji, Michael D. Parkins, Christopher H. Mody

**Affiliations:** Department of Medicine, Cumming School of Medicine, University of Calgary, Pulmonary Diagnostics, South Health Campus, 4448 Front Street SE, Calgary, AB T3M 1M4, Canada; Department of Medicine, Cumming School of Medicine, University of Calgary, Calgary, AB, CanadaDepartment of Microbiology, Immunology and Infectious Diseases, University of Calgary, Calgary, AB, Canada; Department of Radiology, Cumming School of Medicine, University of Calgary, Calgary, AB, Canada; Department of Medicine, Cumming School of Medicine, University of Calgary, Calgary, AB, Canada; Department of Medicine, University of Alberta, Edmonton, AB, Canada; Department of Medicine, Cumming School of Medicine, University of Calgary, Calgary, AB, Canada; Deptartment of Pathology and Laboratory Medicine, Cumming School of Medicine, University of Calgary, Calgary, AB, Canada; Department of Medicine, Cumming School of Medicine, University of Calgary, Calgary, AB, CanadaDepartment of Microbiology, Immunology and Infectious Diseases, University of Calgary, Calgary, AB, Canada; Department of Medicine, Cumming School of Medicine, University of Calgary, Calgary, AB, CanadaDepartment of Microbiology, Immunology and Infectious Diseases, University of Calgary, Calgary, AB, Canada; Department of Medicine, Cumming School of Medicine, University of Calgary, Calgary, AB, CanadaDepartment of Microbiology, Immunology and Infectious Diseases, University of Calgary, Calgary, AB, Canada

**Keywords:** Aspergilloma, antifungals, bronchoscopy, fungal ball, mycetoma

## Abstract

Intracavitary pulmonary aspergilloma is a persistent and life-threatening infection that carries a mortality rate of up to 15%. It occurs when Aspergillus species gain entry to an existing lung cavity. In the absence of definitive treatment, patients may succumb to severe complications such as massive hemoptysis, cachexia, or secondary infections. Aspergillomas often show limited response to antifungal medications, mainly due to insufficient drug concentrations within the cavities. Surgery is frequently the preferred treatment option, but it poses significant risks, and many individuals are ineligible due to underlying health issues. We present the most extensive non-surgical fungal ball cohort to date, managed using an innovative multimodal strategy that combines antifungal therapy before and after bronchoscopic debulking. This was a cross-sectional observational study. For those who cannot undergo surgery, our medical center has pioneered a multimodal approach to aspergilloma resection. This approach combines bronchoscopic endoscopy with antifungal therapy and has been applied successfully to more than 18 patients that are presented in this series. The median age of the cohort was 58 years (range: 32–73), with an equal sex distribution. The mean percent predicted FEV_1_ was 65.3%. The mean follow-up duration was 3.6 years (range: 0.5–10 years). The cohort receiving antifungals systematically prior to debridement showed a reduction of the pre-existing cavity (40.38 mm *versus* 34.02 mm, *p* = 0.021). Across the 18 patients during the follow-up period, 94% remained recurrence-free (defined by symptoms and radiology). Our study fills a critical knowledge gap regarding the significance of initiating antifungal treatment before bronchoscopic debulking and presents a viable approach in these cases for which there is a current unmet therapeutic need.

## Plain Language Summary

The use of both medical and interventional methods to treat difficult fungal masses: A collection of cases showing efficacy for patients who can’t undergo surgery

Intracavitary pulmonary aspergilloma is a serious and potentially deadly infection with a death rate of up to 15%. It happens when certain types of fungi invade existing lung cavities. Without proper treatment, patients may experience severe complications like heavy bleeding from the lungs, weight loss, or other infections. Traditional antifungal medications often don’t work well because they can’t reach high enough concentrations in the cavities. Surgery is usually the best option, but it’s risky and not possible for many due to other health problems. Our study introduces a new way to treat aspergilloma without surgery. We’ve treated a significant number of patients using a combination of antifungal drugs and a procedure called bronchoscopic debulking. This involves removing the fungal growth using a thin tube inserted through the airways. Our research involved observing 18 patients treated this way. They were mostly middle-aged, with equal numbers of men and women. Their lung function was moderately impaired, and we followed them for an average of 3.6 years. We found that giving antifungal drugs before the debulking procedure helped reduce the size of the cavities. After treatment, almost all patients remained free of symptoms and signs of recurrence. This study highlights the importance of starting antifungal therapy before bronchoscopic debulking and offers a promising option for patients who can’t have surgery.

## Background

Intracavitary pulmonary fungal balls, most commonly associated with an aspergilloma, represent a persistent fungal infection with a high mortality rate if untreated.^1^ Pulmonary fungal balls develop when *Aspergillus* spp or other molds inhabit an existing lung cavity that has formed due to various disease processes.^1^ This leads to a prolonged inflammatory reaction and the formation of biofilms, making it resistant to the body’s defenses.^2^ Complications such as severe bleeding from the lungs (massive hemoptysis), malnutrition, or secondary infections can ultimately lead to death.^1^

Aspergillomas typically show limited response to antifungal treatments. Oftentimes, they require long-term suppressive therapy, with varying clinical outcomes due to inadequate antimicrobial concentrations within the cavities, which may contribute to azole resistance.^3^ Despite best efforts, studies of patient groups suggest response rates of approximately 50% for itraconazole or voriconazole when used alone as medical therapy.^4–7^ Surgical resection is the preferred treatment,^8,9^ but it is often not feasible due to compromised lung function and the presence of other health conditions.^10^ The best management strategy for aspergilloma remains an unmet need in this specific patient population. To address this gap, our group initially published a case series detailing aspergilloma resections through debulking by endoscopy^11^ and has recently expanded to introduce a multimodal protocol.^12^ In this report, we enhance our debulking protocol by presenting a practical multimodal therapeutic approach that combines bronchoscopic resection with antifungal treatment. The reporting of this study adheres to the CARE (CAse REport) checklist (see Supplemental Material).^13^

## Case presentations

This was a cross-sectional observational study. All patients (*n* = 18) who underwent endoscopic resection of a lung fungal ball between November 2010 to January 2023 at the Foothills Medical Centre (ethical approval REB23-0150) were reviewed. All patient details were de-identified. Patients were referred to the interventional pulmonary medicine group for consideration of aspergilloma debulking after being deemed non-surgical candidates. Clinical characteristics, co-morbidities, and outcomes were collected from detailed chart audits. The initial cohort (i.e. those without pre-debridement antifungal treatment, subjects 1–8) were compared to those who received antifungal therapy following pre- and post-debridement.

Demographics of the 18 patients are detailed in Table 1. The median age of the cohort was 58 years (range: 32–73), with an equal sex distribution. The mean percent predicted (pp)FEV_1_ was 65.3%. No triggering etiologies were identified in three participants (17%). All patients were symptomatic at the time of bronchoscopy, including refractory cough, hemoptysis, and/or weight loss. The mean size of the aspergillomas prior to resection was 12.1 mm (length) by 23.4 mm (width), as measured on computed tomography. The majority of patients (*n* = 16/18, 89%) were culture positive for a causative mold. *Aspergillus.* sp [including *Aspergillus fumigatus* (*n* = 9), *Aspergillus terreus* (*n* = 1), *Aspergillus niger* (*n* = 1), *Aspergillus versicolor* (*n* = 1) and one *Aspergillus*. sp unable to be speciated] and/or a positive galactomannan was present in 14 patients (78%). Two patients (11%) had a fungal ball secondary to *Pseudallescheria boydii*, identified by culture positivity. Eleven patients (61%) had a concomitant bacterial or mycobacterial pathogen identified by culture.

**Table 1.** Demographics and clinical characteristics of subjects at time of first bronchoscopy.

Patient	Age (years)	Sex	Cause/Inciting event	ppFEV_1_	Pre-procedure (months)^a^	Pre-Cavity (mm)^a,b^	Pre-mycetoma (mm)^a,b^	Sedation ventilation	Complications	Bronchoscopy cultures	LOS (days)	Post-Procedure (months)	Post-cavity (mm)^b^	Post-mycetoma (mm)^b^	Follow-up duration (months)
1	63	F	Idiopathic	–	–	45 × 38 × 39	39 × 34 × 34	GA, Rigid	Intubation	*A. fumigatus*	14	Itra (12)	47 × 44 × 53	0	72
2	67	M	Idiopathic	125	Itra (0.5)	33 × 28 × 21	17 × 18 × 17	GA, ETT	Transient hypoxia	*P. boydii*	5	Vori (6)	28 × 27 × 28	0	84
3	67	F	NCFB	45	Itra (0.5)	31 × 49 × 38	27 × 43 × 31	GA, rigid	Transient NIPPV	*Aspergillus. sp*, MRSA	3	Vori (6)	30 × 43 × 53	0	120
4	63	F	Post-infection	58	Itra (1)	39 × 46 × 42	16 × 12 × 11	GA, ETT	Sepsis, ARDS	*A. fumigatus*	48	Capsofungin (14)	48 × 50 × 47	7 × 6 × 5	48
5^c^	50	F	Post-TB	101	–	28 × 45 × 36	23 × 41 × 32	GA, ETT	Transient hypoxia	*H. influenzae*	4	Vori (6)	25 × 30 × 24	10 × 13 × 6	96
6	32	F	Post-TB	41	–	38 × 45 × 46	33 × 42 × 41	GA, ETT	–	*A. fumigatus* *C. freundii*	10	Vori (3)	36 × 44 × 48	8 × 19 × 6	6
7	45	F	Post-TB	63	Vori (1)	25 × 12 × 23	13 × 11 × 12	GA, ETT	–	*A. terreus* *C. glabrata*	1	Vori (6)	17 × 9 × 23	0	63
8	40	M	Post-TB	40	Vori (0.75)	90 × 75 × 140	23 × 25 × 32	CS	–	*A. fumigatus* MAC	–	Vori (6)	92 × 77 × 14	13 × 19 × 15	12
9	66	F	Idiopathic	81	Vori (3)	17 × 13 × 17	17 × 13 × 17	GA, ETT	–	*A. fumigatus* *S. anginosus*	1	Vori (3)	13 × 13 × 14	0	36
10	33	M	Post-Lung transplant CF	67	Vori (9)	82 × 67 × 60	19 × 20 × 20	CS	–	*A. fumigatus, A. versicolor, E. coli*, MRSA, *B. cepacia* complex	–	Nebulized AmphoB, isavuconazole (ongoing)	72 × 60 × 50	0	36
11^c^	44	F	NCFB	54	Vori/Posa (3)	17 × 20 × 20	15 × 17 × 19	CS	–	*P. aeruginosa*	–	Posa (3)	18 × 21 × 22	0	24
12^d^	73	M	COPD	56	Vori (3)	11 × 25 × 17	10 × 20 × 9	CS	–	-	–	Vori (3)	37 × 32 × 42	12 × 11 × 15	24
13	58	M	GPA	67	Vori (12)	19 × 20 × 19	16 × 15 × 13	GA, ETT	–	*A. fumigatus*	1	Vori (3)	15 × 16 × 14	0	24
14	34	F	CF	42	Vori (4)	19 × 35 × 33	11 × 28 × 32	GA, ETT	–	*P. boydii, P. aeruginosa*	–	Inhaled AmphoB (3)	14 × 30 × 25	0	12
15	69	M	Crohn’s	-	Vori (4)	49 × 39 × 30	14 × 20 × 10	CS	–	*A. niger*	–	Vori (3)	58 × 46 × 35	0	12
16	58	M	Sarcoid	59	Vori (4)	30 × 41 × 31	23 × 34 × 23	GA, ETT	Infection	*A. fumigatus*	–	Vori (ongoing)	39 × 34 × 40	0	6
17^d^	66	M	Empyema	104	Vori (5)	51 × 42 × 45	20 × 13 × 13	GA, ETT	–	–	–	Vori (ongoing)	74 × 44 × 56	0	6
18	47	M	BMT	41	Vori (4)	50 × 55 × 56	15 × 15 × 35	GA, ETT	–	*A. fumigatus, M. gordonae, K. oxytoca*	–	Vori/Posa (ongoing)	2 × 3 × 4	0	18

a Indicates prior to endoscopic debridement.

b Dimensions as measured on CT scan (length × width × height)

c Indicates subjects with radiographic imaging consistent with mycetoma but fungal culture negative.

d Indicates subjects with positive galactomannan by bronchoscopy but fungal culture negative.

ARDS, acute respiratory distress syndrome; BMT, bone marrow transplant; CF, cystic fibrosis; COPD, chronic obstructive pulmonary disorder; CS, conscious sedation; ETT, endotracheal tube; GA, general anesthetic; GPA, Granulomatosis with polyangiitis; IBD, inflammatory bowel disease; Itra, itraconazole; LOS, length of hospitalization stay; m, months; NCFB, non-cystic fibrosis bronchiectasis; Posa: posaconazole; Vori, voriconazole; TB, tuberculosis; w, weeks.

All patients underwent an initial diagnostic bronchoscopy. Subsequently, 11 (61%) patients underwent endoscopic debridement of the fungal ball in the operating room under general anesthesia using flexible bronchoscopy through an endotracheal tube, The remainder were completed in the bronchoscopy suite under conscious sedation (*n* = 5/18, 28%) or by rigid bronchoscopy under general anesthetic in case of potential bleeding (*n* = 2/18, 11%).

The initial cohort of patients (*n* = 8, 2010–2013) received systemic antifungal therapy for a minimum of 3 months following debulking but not prior. However, after complications in the initial cohort were observed,^11^ all subjects were given a minimum of 3 months of preconditioning systemic antifungal treatment prior to debulking. All tolerated antifungal therapy except for one patient who discontinued treatment after 3 weeks due to systemic side effects. Voriconazole, at a dose of 300 mg orally twice daily, was the most common antifungal used (*n* = 12/18, 66.7%). Following endoscopic debridement of the mycetoma, the majority of post-procedure complications and prolonged length of stay were among those in the initial cohort before we began to use antifungal preconditioning. Complications included transient hypoxia (11%), acute respiratory distress syndrome (5.5%), intubation and mechanical ventilation (5.5%) and non-invasive ventilatory support (5.5%). During the use of consolidative antifungals, only one subject experienced a complication 3 weeks following debulking and required a short course of outpatient oral antibiotics for a bacterial super-infection. The length of hospitalization was significantly longer in the initial cohort than those with multimodal therapy (12.1 days *versus* 0.2 days, respectively, *p* = 0.026).

The mean follow-up duration of the cohort was 3.6 years (range: 0.5–10 years). No patients died, and only one required critical care support following the procedure. Across the 18 patients during the follow-up period, 94% remained recurrence-free (defined by symptoms and radiology). Only one individual, a 33-year-old male with a double lung transplant for cystic fibrosis, presented with a recurrent aspergilloma 3 years after original debulking (with partial radiological resolution of the cavity as defined by smaller size in between episodes). The mean size of the aspergillomas following endoscopic resection was smaller compared to initial bronchoscopy at 2.78 mm (*p* < 0.0001). Five patients (28%) did have a small residual persistent fungal ball within the pre-existing cavity despite debulking and antifungal therapy. While there were no differences in the residual cavity pre-and post-bronchoscopy (37.44 mm *versus* 36.94 mm, *p* = 0.87), when partitioned by the cohort receiving antifungals systematically prior to debridement (i.e. 2013 onwards), there was a difference in reduction of the pre-existing cavity (40.38 mm *versus* 34.02 mm, *p* = 0.021). All subjects, apart from the one with recurrence, had symptomatic improvement following therapy across the follow-up period.

## Discussion

Our cohort is the largest reported cohort of non-surgical fungal balls treated with a novel multimodal approach of antifungal therapy pre- and post-bronchoscopic debulking. We have demonstrated safety and tolerability across the majority of subjects. Importantly, our study addresses a key knowledge gap around the role of conditioning antifungal therapy pre-bronchoscopic debulking, where we showed significantly lower length of hospitalization and radiographic improvement. We prescribe systemic antifungals for 3 months, which we acknowledge was selected pragmatically as no literature currently exists to guide duration of this therapy before the procedure to decrease burden of disease and reduce the theoretical risk of soiling the healthy lung during debridement. Accordingly, none of our post-2013 cases (i.e. those who received systemic antifungals preceding and following bronchoscopy) have required embolization before or following removal. Following the procedure, subjects are given consolidative therapy with antifungals for an additional 3 months. While side effects have been reported with azoles,^14^ we demonstrate effectiveness with further reduction in the pre-existing cavity following removal of the fungus ball and antifungal therapy, which may further attenuate recurrence risk.

We acknowledge several limitations of our single-center retrospective study. Our sample size was underpowered, given the small number of patients, but it provides the framework for future, larger multi-center studies. Bronchoscopic debridement appears to be effective, but the optimal duration of antifungal therapy pre- and post-intervention is unknown, and adverse effects warrant consideration in this context. Further studies evaluating candidates for shorter periods of antifungals are required. Our analysis did not account for other forms across the spectrum of Aspergillus lung disease, such as chronic invasive aspergillosis—which may be at higher risk of complications post-procedure. Finally, our analysis does not account for confounders such as co-infections observed with the fungal balls, and further investigation of these complex interkingdom communities in chronic infections is required.^15^

## Conclusion

Taken together, our findings suggest that multimodal therapy with preconditioning and consolidating antifungals and endoscopic debridement in the right patient population is highly effective and has a favorable safety profile. Moreover, our bronchoscopy technique is an attractive option for those individuals with contraindications to general anesthesia or in centers where the availability of an operating room is limited. Significant clinical and radiographic improvements and cure in 94% were observed in non-surgical candidates who had previously been offered the inefficient regimen of salvage azole therapy only. Given the increasing prevalence of COVID-19-associated pulmonary aspergillosis^16^ and likely more to come, this treatment ought to be considered for potential curative intent in those deemed non-surgical and as an alternative to reduce costs and patient morbidity and improve quality of life.

## Supplemental Material

sj-pdf-1-tar-10.1177_17534666241255203 – Supplemental material for Combined medical-interventional approaches for the management of complex fungal balls: a case series as a viable alternative in non-surgical patients Supplemental material, sj-pdf-1-tar-10.1177_17534666241255203 for Combined medical-interventional approaches for the management of complex fungal balls: a case series as a viable alternative in non-surgical patients by Elaine Dumoulin, Christina S. Thornton, John H. MacGregor, Alain Tremblay, Chrystal Chan, Paul R. MacEachern, Margaret M. Kelly, Ranjani Somayaji, Michael D. Parkins and Christopher H. Mody in Therapeutic Advances in Respiratory Disease

## Declarations

**Ethics approval and consent to participate:** The study was approved by the Health Research Ethics Board at the University of Calgary (REB23–0150; Calgary, Alberta, Canada). Given the retrospective nature of the study, a waiver of consent was granted for patient information.

**Consent for publication:** Based on the retrospective nature of this series dating over 10 years, ethical approval was granted for waiver of consent for these cases.

**Availability of data and materials:** The datasets used or analyzed during this study are available from the corresponding author on reasonable request.
